# Sensorimotor Integration in Dyslexic Children under Different Sensory Stimulations

**DOI:** 10.1371/journal.pone.0072719

**Published:** 2013-08-16

**Authors:** André R. Viana, Milena Razuk, Paulo B. de Freitas, José A. Barela

**Affiliations:** 1 Graduate Program in Human Movement Sciences – Institute of Physical Activity and Sport Sciences, Cruzeiro do Sul University, São Paulo, São Paulo, Brazil; 2 Institute of Biosciences, Sao Paulo State University, Rio Claro, São Paulo, Brazil; University of Chicago, United States of America

## Abstract

Dyslexic children, besides difficulties in mastering literacy, also show poor postural control that might be related to how sensory cues coming from different sensory channels are integrated into proper motor activity. Therefore, the aim of this study was to examine the relationship between sensory information and body sway, with visual and somatosensory information manipulated independent and concurrently, in dyslexic children. Thirty dyslexic and 30 non-dyslexic children were asked to stand as still as possible inside of a moving room either with eyes closed or open and either lightly touching a moveable surface or not for 60 seconds under five experimental conditions: (1) no vision and no touch; (2) moving room; (3) moving bar; (4) moving room and stationary touch; and (5) stationary room and moving bar. Body sway magnitude and the relationship between room/bar movement and body sway were examined. Results showed that dyslexic children swayed more than non-dyslexic children in all sensory condition. Moreover, in those trials with conflicting vision and touch manipulation, dyslexic children swayed less coherent with the stimulus manipulation compared to non-dyslexic children. Finally, dyslexic children showed higher body sway variability and applied higher force while touching the bar compared to non-dyslexic children. Based upon these results, we can suggest that dyslexic children are able to use visual and somatosensory information to control their posture and use the same underlying neural control processes as non-dyslexic children. However, dyslexic children show poorer performance and more variability while relating visual and somatosensory information and motor action even during a task that does not require an active cognitive and motor involvement. Further, in sensory conflict conditions, dyslexic children showed less coherent and more variable body sway. These results suggest that dyslexic children have difficulties in multisensory integration because they may suffer from integrating sensory cues coming from multiple sources.

## Introduction

Dyslexia has been characterized by a failure to attain the expected literacy skills for a given age, despite adequate intellectual ability and sufficient educational provision (e.g., [1,2]). However, difficulty in literacy acquisition is only one of many symptoms experienced by dyslexic individuals. Studies have shown that dyslexic children and adults present inconsistent and highly variable motor performance during postural control tasks [1,3–5] as well as during gait [3]. Moreover, significant differences on postural control performance between dyslexic children and age- and gender-matched controls were also reported in previous studies involving dual task. These studies clearly demonstrated that the postural control of dyslexic children was highly impaired by addition of a second task that increases attentional demands [5–7].

These evidences indicate a much larger scope of symptoms, which question the specificity of this disorder and its relation only to literacy competence. More important, however, is that these evidences have intrigued and inspired research in different domains with the goal of uncovering the underlying neural mechanisms related to the impoverished literacy acquisition as well motor performance. For instance, it has been suggested that dyslexic individuals may suffer from a mild cerebellar deficit [2,8] that would impair the ability of these individuals to perform tasks with automaticity [9]. Lack of automaticity in performing tasks such as reading, writing, walking, and maintaining upright stance would require much more conscious efforts [9] in addition to affecting performance. Despite differences in automaticity being a compelling suggestion, it is necessary to further explain “automaticity of what”? We have recently demonstrated using the moving room paradigm (i.e., paradigm used to observe the effect of dynamic changes in the visual environment on postural sway) that dyslexic children couple sensory information to body oscillation slightly different than their peers in upright stance while visual information was manipulated [10]. Based upon these results, we further suggested that motor performance of dyslexic individuals would be affected by their difficulty to transform multiple sensory cues available in relevant information to perform purposeful task in an automatic manner. Specifically, to use multiple sensory cues to implement proper motor activation required to maintain upright stance [10].

As in many other tasks, postural control requires a complex relationship between sensory information and motor activity [11], which depends upon the automatic identification of current body dynamics, such as body position and sway velocity, and organization of appropriate muscular activation aiming to maintain or achieve a desired postural orientation. This process seems to be accomplished in many cases automatically [12,13], although may be consciously disrupted [14]. Despite being able to use sensory information to estimate body position and sway magnitude and velocity, dyslexic children used sensory information to produce body sway which was characterized by more variability, suggesting that they may have difficulties to couple sensory information and motor activity [10]. However, sensory information in this referred study occurred by manipulating the surrounding environment as dyslexic children stood upright inside a moving room. Considering that many studies have suggested that dyslexia might be related to visual system deficits, i.e., the magnocellular hypothesis [15], our results still could be related to the inappropriate inputs originating from this specific sensory system.

Jeka and colleagues [16–18] have developed a experimental paradigm that mimics the moving room manipulation by asking individuals to touch lightly (less than 100 g or 1 N of applied force) their fingertip to a moveable plate during upright stance. In doing so, individuals sway coherently to the plate oscillation without consciously being aware of doing it. Such as experimental strategy was implemented to examine the relationship between additional somatosensory information (i.e., touch) and body sway in children of different ages [19] and it seems convenient to uncover any difference in the use of somatosensory cues in dyslexic children. Moreover, combining touch and vision in different experimental conditions, it is possible to examine how dyslexic children would integrate sensory stimuli coming from different sources and, consequently, uncover possible differences in multisensory integration. Therefore, the aim of this study was to compare the performance of the postural control system and the relationship between sensory information and body sway, with vision and touch being manipulated independent and concomitantly, between dyslexic and non-dyslexic children. We hypothesized that dyslexic children would sway with larger magnitude than non-dyslexic children and would present a weaker and more variable relationship between body sway than non-dyslexic children, especially in conflicting sensory conditions.

## Materials and Methods

### Ethics Statement

All children’s participation in the study was conditioned to the permission given by the parents through signature of an informed consent form. Both this study and the informed consent form were approved by the local Institutional Review Board (University of Cruzeiro do Sul Ethics Committee / Protocol #UCS161/2009).

### Participants

Thirty dyslexic (11.2 ±1.4 year-old, 17 boys and 13 girls) and thirty age- and gender-matched non-dyslexic children (11.1 ±1.5 year-old, 17 boys and 13 girls), with no reading difficulties and unknown neurological, orthopedic, and musculoskeletal conditions participated in this study. Dyslexic children were recruited from the Brazilian Dyslexic Association and from the Institute of Specialization in Clinical Speech in which they were referred to a complete evaluation of their dyslexia including neurological, psychological, and phonological capabilities. Children were evaluated regarding decoding letters, reading and comprehension text (PROLEC test) and phonological awareness (CONFIAS test). PROLEC is a validated Brazilian test [20] used to classify the children’s reading level (no reading, moderate, and severe problem). Twenty-eight children were classified as moderate and two as severe. CONFIAS is also a validated Brazilian test [21] used to assess the writing skill level. As inclusion criteria, children should show writing skill performance at least one standard deviation below from the expected mean score. In addition, children were also classified with normal intelligence quotient (WISC-III), scores raging from 90 to 120, and showed no signs of hyperactivity (DSMV-IV). Based upon the mentioned tests, children were characterized as mildly dyslexic, with the exception of two of them who were classified as severely dyslexic.

### Procedure

Each participant was brought to the laboratory and, after a brief period of adaptation, was prepared for a single-day experimental session. Participants were asked to stand upright as quiet as possible inside of a moving room. This moving room consists of three walls and a ceiling (2 m long x 2 m wide x 2 m high), with wheels placed on linear rails so that it could be moved backward and forward. The room was moved by a servomotor system composed of a linear guide (Ottime, model PL6-90C-LD-MT-RC), a stepper motor (Ottime, model SM3452808), and a motor driver (Ottime, model MBD-8080DC) and controlled by a specific software (Motion Planner 4.3). The walls were white with black stripes, creating a pattern of 42 cm wide vertical white and 22 cm wide vertical black stripes. Two 20-watt triple tube compact fluorescent light bulbs were placed horizontally on the backside of the ceiling and directed to the front wall to maintain the same luminosity throughout the data collection. Inside of the moving room and in front of the participants, it was placed a moving touch plate. This touch plate consists of a round metal plate (diameter of 4 cm), connected to a load cell, and fixed inside of a metal box. This box was mounted on a linear guide (Ottime, model PL3-30C-LE-MT-R000), which can be moved, back and forth, also by a servomotor system, constituted by a stepper motor (Ottime, model SM23 SSF1192108), a motor driver (Ottime, model MBD-2278AC), and controlled by a specific software (Motion Planner 4.3). The load cell was connected to an amplifier (EMG System Brazil) and provided information about the vertical force applied on the metal plate. Force data were collected using an analogical/digital converter (OPTOTRAK ODAUII - Northern Digital Inc.), at a sampling rate of 100 Hz, and displayed in real time during acquisition. The whole touch plate, mounted on a tripod, was adjustable to be positioned right in front of the participant and at hip height.

In the trials in which the room and/or the touch plate was moved, continuous backward and forward oscillation was created at frequency of 0.2 Hz, amplitude of 0.3 cm, and peak velocity of 0.38 cm/s. In the trials that vision was allowed, participants were asked to fixate a target (round white paper with 3 cm of diameter) placed on the moving room frontal wall, distant 1.5 m and at eye level. In the trials that the light touch was allowed, participants were asked to place their right index fingertip on the touch plate and maintain it still. Applied force was limited in 1 N and the current force value was provided during the trial. In case that applied force level went over 1 N, participants were asked to decrease applied force without losing contact with the surface. Nevertheless, episodes that vertical force was over 1 N were very rare.

An OPTOTRAK (Certus - Northern Digital Inc.) IRED emitter was placed on the participant’s back (at approximately the 8^th^ thoracic vertebra level), a second IRED emitter was placed on the front wall of the moving room, and a third one was placed on the moveable basis of the touch plate. These markers provided information about participant’s trunk sway and the moving room and touch plate displacement in the anterior–posterior, medial-lateral, and vertical directions, with a sampling rate of 100 Hz.

Each participant performed 5 trials, each one lasting 60 seconds. In the first trial, moving room and touch bar remained stationary and participants were asked to maintain upright stance with eyes closed and arms hanging aside their body (baseline). In the second trial, visual information was manipulated by using a moving room, where participants were asked to maintain upright stance fixating the target placed in front of them and arms hanging aside their body (moving room condition). In the third trial, somatosensory information was manipulated by using a moving bar. In this condition, the participants were asked to maintain upright stance with eyes closed touching the bar with their right index finger (moving bar condition). The order in which these trials (2^nd^ and 3^rd^ trials) occurred was randomized. Finally, in the fourth and fifth trials, vision and touch conditions were combined to enhance sensory conflict. Participants were exposed to the moving room while touching the plate that remained stationary (moving room and stationary bar condition); and touching a moving bar while the room was stationary (moving bar and stationary room condition). The order in which these trials occurred was also randomized.

Since it has been shown that prior knowledge about the room’s movement influences coupling between visual information and body sway [12,14], participants were not told about the visual and touch manipulation. At the end of the experiment, participants were asked whether or not they noticed anything unusual about the room or the touch plate. None of the participants, dyslexic and non-dyslexic children, stated that they were aware that the room or the touch plate was moving.

### Data analysis

Since the room or the touch plate was oscillated in the anterior–posterior direction, the analyses focused only on this direction. Trunk sway, moving room and moving bar position time-series in the anterior–posterior direction were filtered with a 4^th^ order, zero-lag, digital Butterworth filter with a cutoff frequency set at 4 Hz. Two groups of dependent variables were calculated, one related to the body sway magnitude and another related to the relationship between moving room/bar and body sway. The first analysis examined the participant’s behavior through the variable mean sway amplitude, obtained by calculating the standard deviation of the detrended trunk sway signal. The mean sway amplitude was used to examine the average performance of the postural control system, indicating body sway magnitude as children maintained the upright stance position as quiet as possible. The second group of variables provided information about the relationship between the moving room or the moving bar and trunk sway. The relationship between visual and/or touch information and body sway was examined by three variables: coherence, position variability and velocity variability. Spectral analysis was performed for each trial by computing the individual Fourier transforms of the trunk sway and moving room or touch plate time-series. Based upon these transforms, coherence between body sway and the visual/touch stimulus was computed at the stimulus driving frequency (0.2 Hz). Coherence indicated how strongly body sway was coupled to the visual/touch stimulus with values close to 1 (zero) indicating that the signals demonstrate strong (no) dependency between them.

Position and velocity variability of the trunk sway was computed as the standard deviation of the trunk sway after the deterministic response to the sensory drive is subtracted (cf. [22]). For the position and velocity trajectories, the Fourier transform was computed, the value of the transform at the stimulus frequency was removed, and then, the inverse transform was computed, resulting in a ‘residual’ position and velocity trajectories. Position and velocity variability was computed as the standard deviation of the residual position or velocity trajectory, respectively, and indicate body sway magnitude and velocity in frequencies other than the one of the visual/touch information manipulation. In those trials that touch was allowed, applied force was also filtered by a 4^th^ order, zero lag, digital Butterworth filter with a cutoff frequency set at 5Hz. After this procedure, average of vertical force applied to the bar was obtained for each trial.

After data normality and homogeneity of variance assumptions were tested and fulfilled, univariate (ANOVAs) and multivariate analyses of variance (MANOVAs) were employed. In order to test group (dyslexic and non-dyslexic) effect in maintaining upright stance with eyes closed and no touch (baseline condition), one ANOVA with mean sway amplitude as dependent variable was employed. In the trials with visual and touch manipulation, two ANOVAs and one MANOVA [group (dyslexic and non-dyslexic) and condition (vision and touch manipulation)], with the last factor treated as repeated measures, were carried out. The dependent variables for the ANOVAs were mean sway amplitude and coherence. The dependent variables for the MANOVA were position and velocity variability values. Similarly, two ANOVAs and one MANOVA (group and condition) were employed for the trials with sensory conflict (trials 4 and 5). The dependent variables were the same as the previous analyses. Finally, one ANOVA was used to test group and condition, having as dependent variable the amount of vertical force applied. In all analyses, condition was treated as repeated measure. Appropriate follow-up univariate analyses were performed, when applicable, with the overall significance level set at .05. All analyses were performed using SPSS (SPSS for Windows 10.0).

## Results

### Body sway magnitude

#### Baseline trial

**Figure 1:**
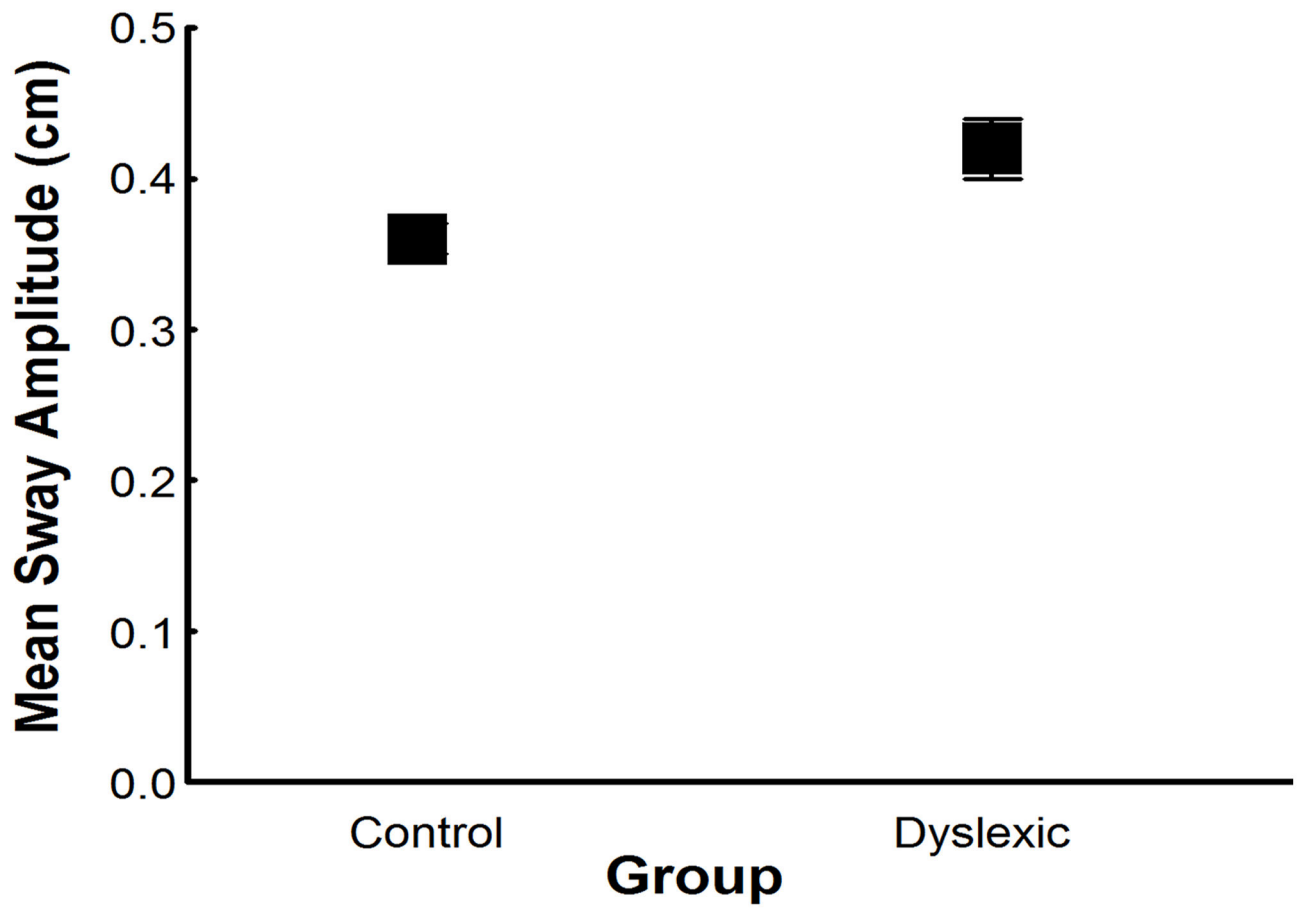
Mean sway amplitude. Trunk sway in the anterior–posterior direction for both groups with eyes closed and without touch. Error bars represent standard error.

Figure 1 depicts mean sway amplitude values for both groups in the trial in which children maintained upright stance with no vision. ANOVA revealed an effect of group, F(1,58)=4.56, p<.05, effect size=.073 (ES, partial eta square calculation was used), on mean sway amplitude. Dyslexic children swayed more than non-dyslexic children in the anterior–posterior direction.

#### Vision and touch manipulation

**Figure 2:**
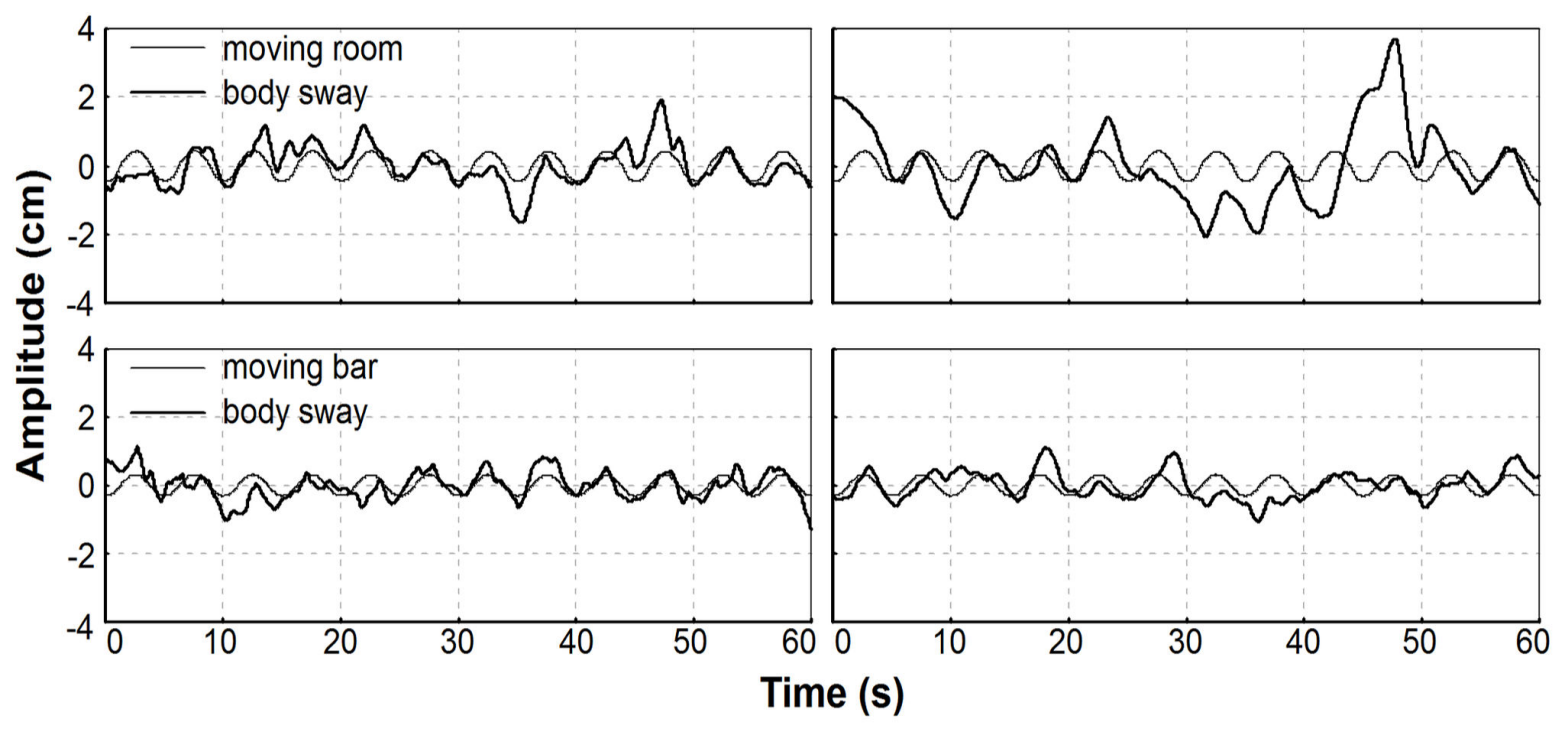
Time-Series. Trunk sway and moving room (top panels) and moving touch plate (bottom panels) displacement time-series for a non-dyslexic (left column) and for a dyslexic (right column) participant in the anterior–posterior direction.

Visual and touch manipulation, from the moving room and the moving bar, induced correspondent body sway in both groups. Figure 2 depicts an exemplar time-series of trunk sway and moving room/touch plate displacement for a non-dyslexic and a dyslexic child. The overlaid time series shows that the trunk adopts the frequency of the moving room/touch plate along the trial.

**Figure 3:**
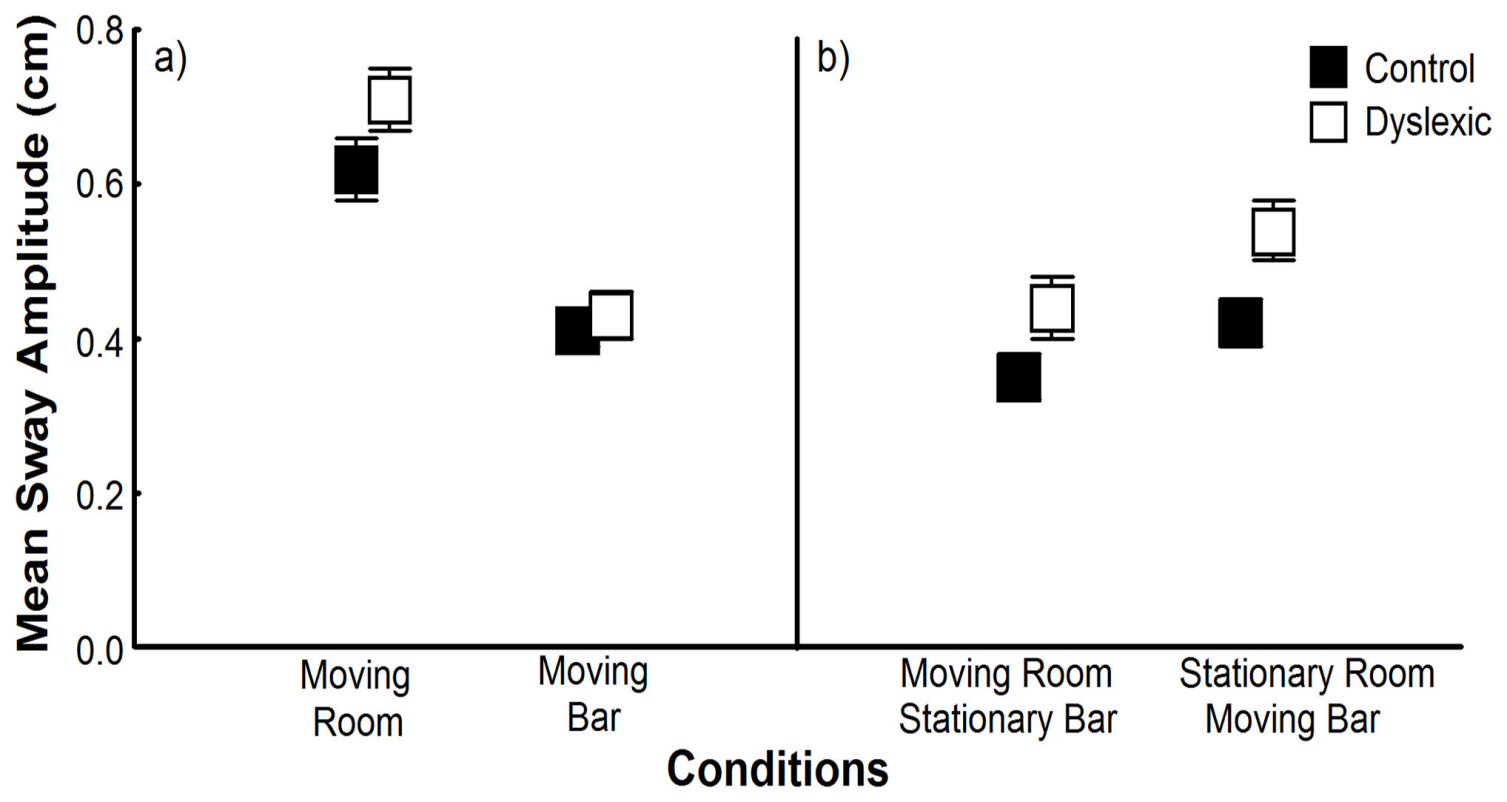
Mean sway amplitude. Trunk sway in the anterior–posterior direction for both groups in the trials in which room or bar was moved (a) and in the trials with conflicting sensory information (b). Error bars represent standard error.

Figure 3 shows mean sway amplitude values for both groups in the trials in which children maintained upright stance inside of the moving room either lightly touching or not touching a moveable surface. In the trials that vision and touch were manipulated independently (Figure 3a), ANOVA revealed effect of group, F(1,58)=4.16, p<.05, ES=.067, and stimulus, F(1,58)=86.19, p<.001, ES=.598. Overall, dyslexic children swayed with larger magnitude than non-dyslexic children and vision (moving room) induced larger sway than touch manipulation. In those trials with sensory conflict (4 and 5 trials), ANOVA also revealed an effect of group, F(1,58)=7.51, p<.05, ES=.115, and stimulus, F(1,58)=17.41, p<.001, ES=.231 (Figure 3b). Independent of the combination of sensory stimulus, dyslexic children swayed with larger magnitude than non-dyslexic children. Mean sway amplitude was larger in the condition in which the moving bar was oscillated and the room remained stationary (stationary room and moving bar condition) compared to the trial in which the moving room was oscillated and the bar remained stationary (moving room and stationary bar condition).

#### Relationship between body sway and sensory information

**Figure 4:**
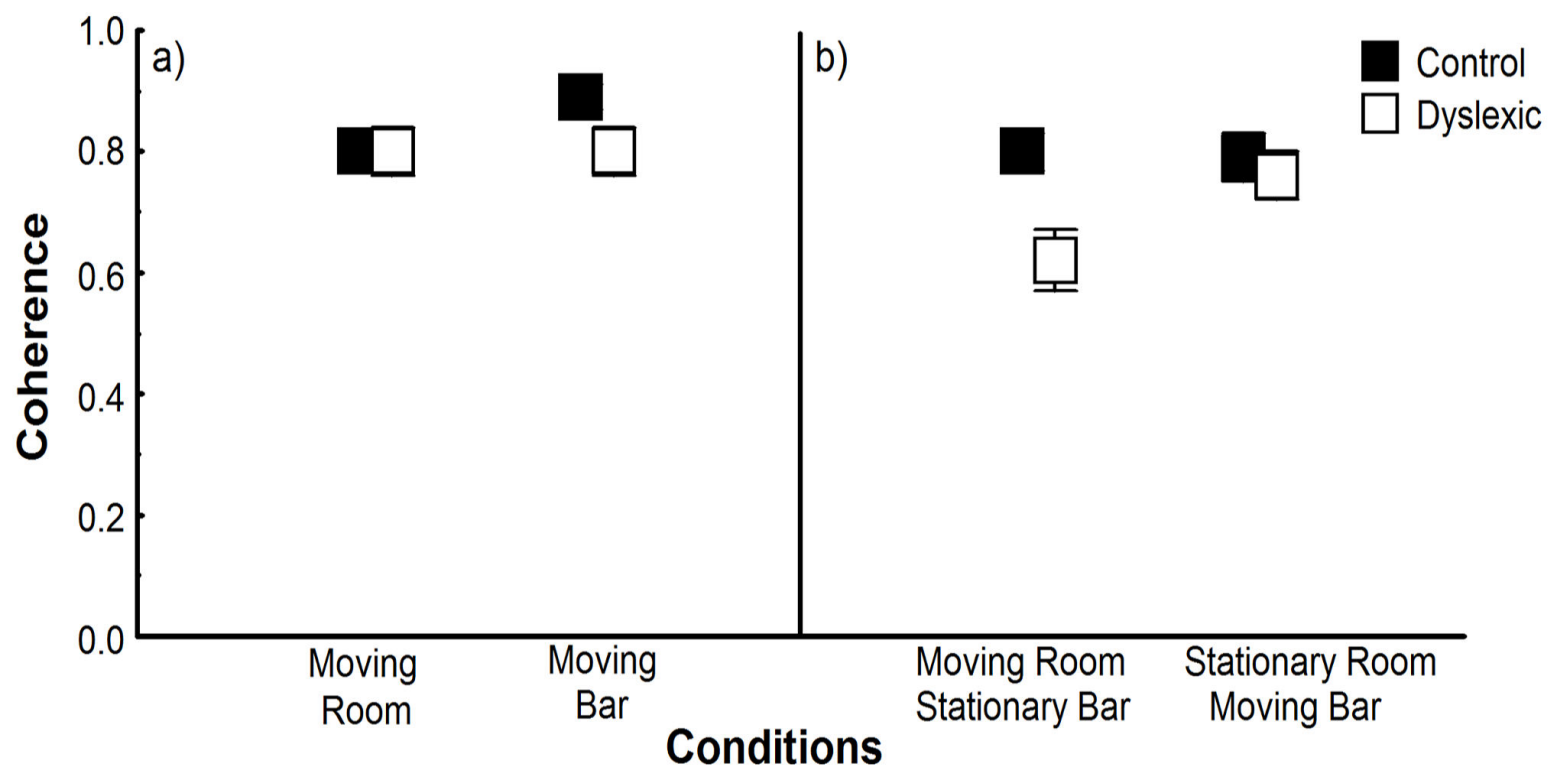
Coherence. Mean coherence between the driving signal and trunks sway for both groups in the trials in which room or bar was moved (a) and in the trials with conflicting sensory information (b). Error bars represent standard error.

The relationship between body sway and visual/touch information was similar between dyslexic and non-dyslexic children and between visual and touch information in those trials in which these stimuli were manipulated independently, but weaker in dyslexic children in those sensory conflicting trials. Figure 4 depicts average coherence values for both groups when vision and touch were manipulated independently and when sensory conflict occurred. ANOVA revealed no main effect of group and stimulus and interaction between factors when vision and touch were manipulated independently (Figure 4a). In those trials with conflicting cues (Figure 4b), ANOVA revealed only effect of group, F(1,58)=5.54, p<.05, ES=.087. Overall, coherence values were lower for the dyslexic compared to non-dyslexic children in situations of sensory conflict.

**Figure 5:**
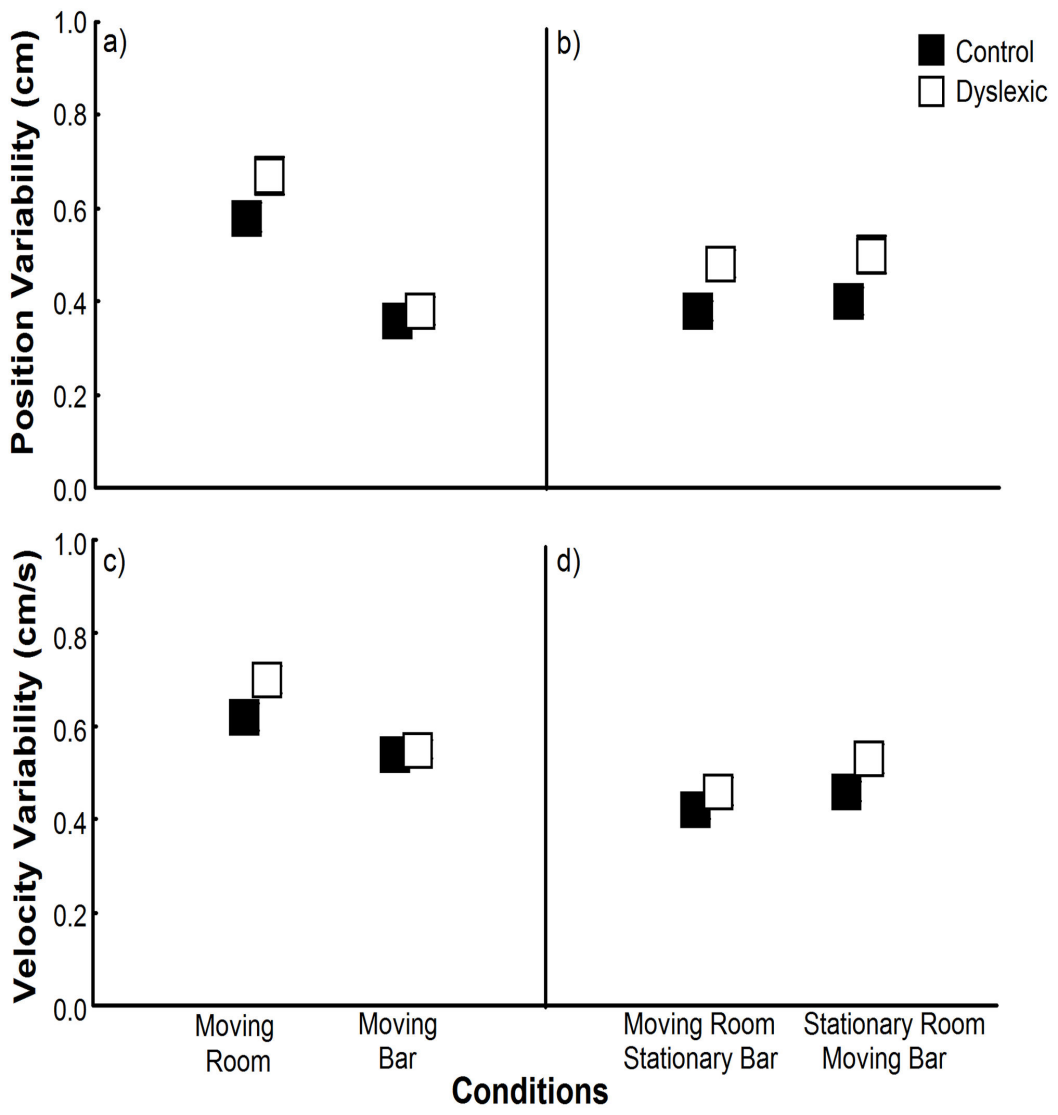
Position and velocity variability. Mean position variability values (a–b) and mean velocity variability values (c–d) for both groups in the trials in which room or bar were moved (a–c) and in the trials with conflicting sensory information (b–d). Error bars represent standard error.

Besides showing weaker relationship between sensory manipulation and body sway than non-dyslexic children, dyslexic children also display more variable body sway than non-dyslexic children. Figure 5 depicts position and velocity variability for both groups and for all conditions. In those trials with independent sensory manipulation (Figure 5a and 5c), MANOVA revealed a main effect of condition, Wilks’ Lambda = 0.39, F(2,57)=44.55, p<.001, and a group by condition interaction, Wilks’ Lambda = 0.85, F(2,57)=5.02, p<.05. Univariate analyses revealed effect of condition on both position, F(1,58)=87.23, p<.001, ES=.601, and velocity variability, F(1,58)=54.37, p<.001, ES=.484, with higher variability for both groups when vision was manipulated compared to touch manipulation. Univariate analyses showed that group and condition interaction occurred only for velocity variability, F(1,58)=9.675, p<.005, ES=.143, revealing that while velocity variability was similar between groups for the touch bar manipulation, dyslexic children swayed with higher velocity variability in the visual manipulation condition compared to non-dyslexic children.

In those trials with conflicting sensory manipulation (Figure 5b and 5d), MANOVA revealed main effect of both group, Wilks’ Lambda = 0.87, F(2,57)=4.13, p<.05, and stimulus condition, Wilks’ Lambda = 0.79, F(2,57)=7.24, p<.005. Univariate analyses revealed effect of group on both position, F(1,58)=7.80, p<.05, ES=.119, and velocity variability, F(1,58)=5.81, p<.05, ES=.091, with higher position and velocity variability for the dyslexic compared to the non-dyslexic children. Univariate analyses also showed effect of condition on velocity variability, F(1,58)=14.322, p<.001, ES=.198. Overall, was observed higher velocity variability for the moving bar and room stationary trial compared to the moving room and stationary bar conditions.

**Figure 6:**
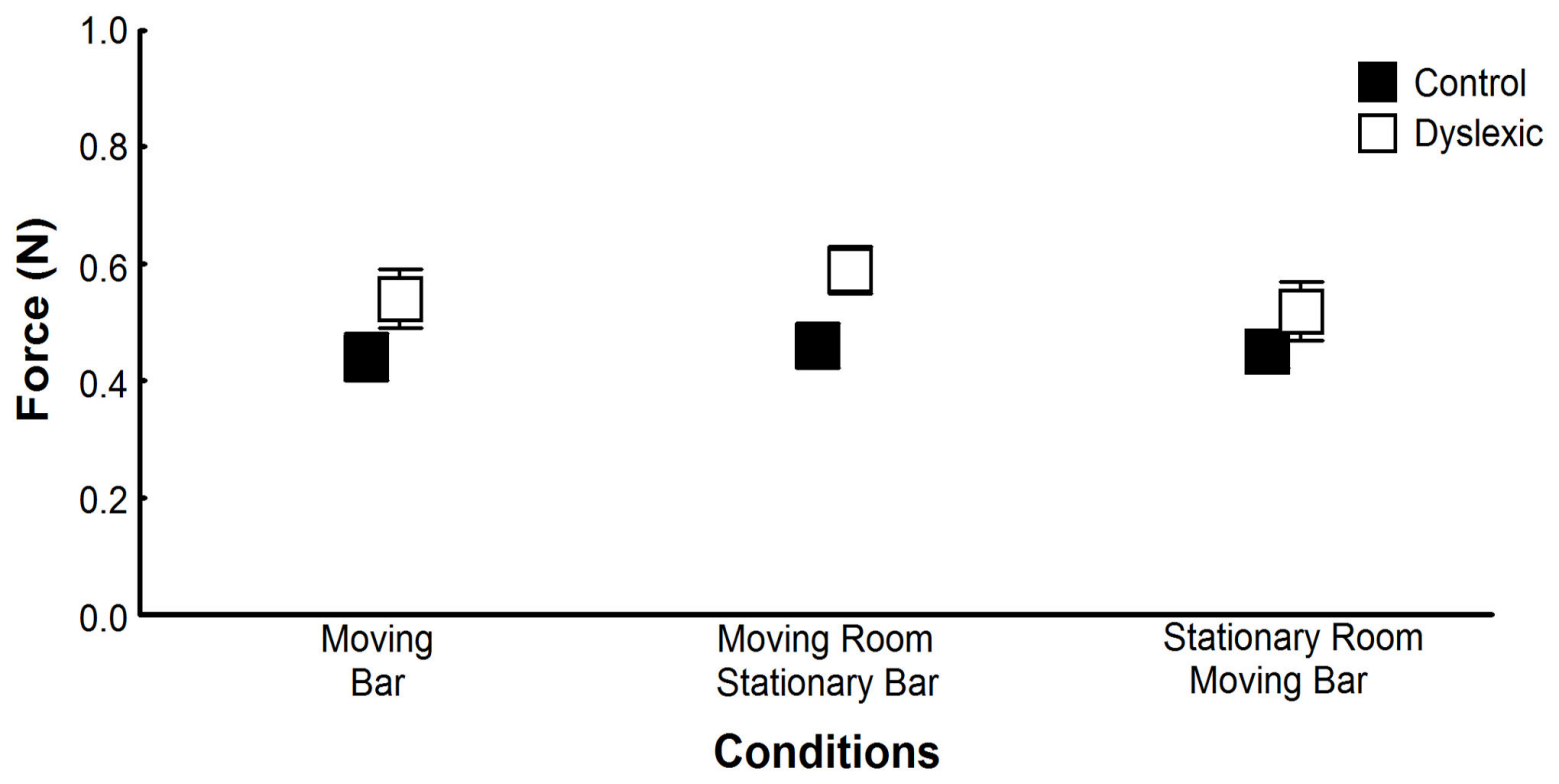
Vertical Fingertip Force. Mean vertical forces applied for both groups in the trials in which participants touched the bar. Error bars represent standard error.

Finally, Figure 6 depicts mean vertical force applied on the touch bar for both groups and all conditions in which touch was allowed. ANOVA revealed only group effect, F(1,58)=4.26, p<.05, ES=.069, showing that despite applying forces below 1N, dyslexic children applied higher magnitude of force than non-dyslexic children during touch conditions.

## Discussion

The aim of this study was to compare the performance of the postural control system and the relationship between sensory information and body sway, with vision and touch being manipulated independent and concomitantly, between dyslexic and non-dyslexic children Our findings indicate that dyslexic children present a poorer performance of the postural control system because they swayed more than non-dyslexic children in situations with no vision and when visual and somatosensory information were manipulated. Also, our findings demonstrate that visual and touch manipulation, due to the moving room and the moving touch bar, induced corresponding body sway in both dyslexic and non-dyslexic children, showing that dyslexic children are able to use information provided by the visual and the somatosensory systems but not similarly to non-dyslexic children when sensory information was manipulated independent and concomitantly. Despite being able to use sensory information to estimate body position and sway magnitude and velocity, the coupling between sensory information and body sway was weaker in dyslexic children in conditions that visual and touch were manipulated concomitantly (i.e., situations of enhanced sensory conflict). In addition, in all conditions that sensory information was manipulated, independent and concomitantly, dyslexic children swayed with more variability compared to non-dyslexic children. Finally, dyslexic children used higher applied force levels compared to non-dyslexic children in those conditions in which touch was required. These results suggest that dyslexic children might have difficulties in coupling sensory information and motor action, especially in multisensory conditions, resulting in poor and more variable motor task performance.

Dyslexic children displayed larger body sway standing upright compared to non-dyslexic children when visual information was absent. This result is not surprising since previous investigations have yet shown that dyslexic children sway more than non-dyslexic children when visual information was present [1,10] or absent [23]. Also, corroborating the finding of our recent study [10], dyslexic children sway more than non-dyslexic children when this sway is driven by an externally imposed vision manipulation. A new finding of the present study is that when somatosensory information is manipulated (i.e., light touch in a moving bar) independent and concomitant with the manipulation of the visual field (i.e., moving room) the larger body sway in dyslexic children persists. Definitely, such larger body sway across all sensory conditions suggests that the postural control system of dyslexic children is more affected by sensory manipulation possibly because of changes in the processing of multisensory information and not only visual information. Consequently, dyslexic children obtain inaccurate information about the surrounding environment and about the relation between neighbor body segments and, therefore, are less efficient in controlling body position in space.

As showed previously, dyslexic children is influenced by visual manipulation as shown by a strong coupling between room movement and body sway [10]. Similarly, the result of the present study indicated that this is also the case for somatosensory information furnished by contacting lightly the fingertip to a movable surface. Such results further indicate that the neural structures responsible for the integration of sensory information and motor commands might be preserved in dyslexic children. Differently than previously observed, our present results indicate no difference in the coupling strength (similar coherence values) between visual information and body sway when comparing dyslexic and non-dyslexic children, with dyslexic children in the present study showing stronger coupling than previously observed. A possible explanation for such results discrepancy to our previous results might be due to difference in stimulus characteristics (smaller amplitude and lower velocity of the moving room). Such stimulus changes were made necessary in order to accommodate the sensory manipulation employed in this study (i.e., moving bar and room with similar oscillation amplitude). In our previous study [10], room movement amplitude was 0.55 cm, while in the present study it was 0.3 cm. This difference in amplitude did not affect non-dyslexic children’s coupling (similar coherence values to the ones previously observed) but affected dyslexic children (i.e., Dyslexic children in this study showed larger coherence during moving room situation than children from our previous study [10]). Another plausible explanation is that dyslexic children, in the present study, were recruited by institutions that make the diagnosis of dyslexia as well as apply interventions to improve the literacy and sensory-motor symptoms of dyslexia. Unfortunately, rigorous control of possible intervention effects on our results was not possible.

Although dyslexic children showed similar coupling strength between sensory information and body sway when visual and somatosensory information was manipulated independently, weaker and more variable coupling was observed in dyslexic children when vision and touch were manipulated concomitantly. Those facts could indicate automatization difficulties, suggested as an underling cause of dyslexia [1,4,9], that would result in poor sensorimotor integration [10]. Our results indicate that weaker coupling is further observed in conditions in which two sensory modalities were manipulated, especially when such manipulation would enhance sensory conflict. As far as we know this is the first study using this experimental protocol in dyslexic children and its findings bring new evidences about the affected multisensory integration in dyslexic children.

Based upon our results, we can suggest that multisensory integration is slightly affected in dyslexic children. Impaired multisensory reweighting was also observed in children with developmental coordination disorder, when vision and touch were manipulated [24]. Moreover, impaired multisensory integration has a negative effect in motor performance such as maintaining upright stance in different sensory conditions. Such suggestion may account for the dyslexic children’s larger sway observed with eyes closed and with vision and light touch manipulation. In the case of this study, dyslexic children different performance would not be due to the use of specific sensory cues coming from a single channel but due to how sensory cues coming from multiple sources are integrated to obtain precise information regarding body position and sway velocity. If this is the case, disruption of performance in many domains, i.e., motor and reading and writing literacy, would have a common cause.

Although such suggestions do not allow us to discard possible sensory system functioning deficits, as the one forwarded due to the magnocelular explanation (e.g., [15]), the mild cerebellar functioning deficit explanation seems to be more suitable to explain some of the underlying neural deficits in dyslexics. A closer look at some of our results regarding the coupling strength between sensory information and body sway (Figure 4b) indicates a quite interesting and promising venue that might support this forthcoming suggestion. Conflicting sensory cues deteriorates coupling strength of both dyslexic and non-dyslexic children, but more dramatically of dyslexic children. Another important evidence regarding difficulties in integrating sensory cues from multiple sources in dyslexic children comes from the observation that these children, although able to follow the instruction of applying forces below 1N, applied higher force levels than non-dyslexic children in all conditions of light touch. If dyslexic children have difficulties in identifying from all the available the most important sensory cues, when possible they attempt to enhance the quality of the ones that would provide reliable information. Recently, Bair and colleagues [25] have showed that children with developmental coordination disorder take advantage of using vision with touch information for standing but only showed sensory reweighting in conditions with large stimulus (visual) amplitude [24]. These results, although related to different population, is another indicative that dyslexic children, which is also a developmental disorder, requires enhanced and robust information to properly integrate the cues coming from multiple sources in order to obtain precise information about body dynamics in maintaining upright stance.

Such suggestion would also be suitable to explain the lateral masking in reading tasks. In this case, when cues are made more distinguished, dyslexic children can improve their reading performance [26]. In the case of the present study, higher level of applied force seems to enhance somatosensory information furnished by touching a surface and, therefore, contributes to postural performance. In conditions that sensory cues cannot be enhanced, dyslexic children’s behavior is affected with lower performance and/or more variability compared to non-dyslexic children. It seems to be the cases when vision and touch information were manipulated independently. Variability was similar for the moving bar when comparing dyslexic and non-dyslexic children, but higher for the moving room condition. Improvement in lowering variability in the touch condition was due to enhance fingertip cues obtained by higher levels of the applied forces by the dyslexic children.

As previously mentioned, dyslexic individuals may suffer from a mild cerebellar deficit [2,8] that would impair the ability of these individuals to perform tasks with automaticity [9]. We have examined such suggestions by investigating how the nervous system integrates sensory cues coming from multiple sources. Clearly, dyslexic children can couple and integrate sensory cues but slightly different than non-dyslexic children. Actually, much of the present findings related to dyslexic children also have been observed for children with other developmental disorders [24,25]. A possible mechanism involved in the sensory integration is the development of an adult-like computational efficiency for multisensory fusion, which is learned and based upon prior estimates in order to furnish the basis for future estimation. Moreover, such control mechanism relies on feedback process with control based on the continuous estimation of body dynamics, which is not fully developed, and more affected by a much noisier motor control system as observed for children with developmental coordination disorder [24] and we might add for dyslexic children as well. For instance, dyslexic children can integrate cues from multiple sources but not as accurately as non-dyslexic children and, therefore, produce motor action with more variability. However, if this is the case, we can speculate that dyslexic children would take advantage of intervention protocols directed to improve sensory integration.

In summary, dyslexic children were influenced as non-dyslexic children by manipulation of visual and somatosensory information producing correspondent body sway. When vision and somatosensory information were manipulated independently, the strength of the sensorimotor coupling was similar in dyslexic and non-dyslexic children. However, in those conditions that vision and somatosensory cues were manipulated concomitantly, coupling strength was weaker and more variable in dyslexic compared to non-dyslexic children. Moreover, dyslexic children applied higher magnitude of fingertip force in the moving bar than non-dyslexic children. Based upon these results, we suggested that dyslexic children experience difficulties in sensorimotor integration due to mild cerebellar deficit.
